# Changes in Foot Biomechanics During Pregnancy: Associations With Plantar Pressure, Low Back Pain and Daily Function in Taiwanese Women

**DOI:** 10.1002/jfa2.70094

**Published:** 2025-11-03

**Authors:** Meei‐Ling Gau, Hui‐Mei Hsu, Li‐Li Chen, Tzu‐Ling Chen, Wan‐Lin Pan

**Affiliations:** ^1^ Department of Nurse‐Midwifery and Women Health National Taipei University of Nursing and Health Sciences Taipei Taiwan; ^2^ School of Nursing National Taipei University of Nursing and Health Sciences Taipei Taiwan

**Keywords:** functional impairment, low back pain, plantar pressure, postural balance, pregnancy

## Abstract

**Background:**

Longitudinal data on pregnancy‐related changes in plantar loading and balance are limited, particularly among Asian populations. This study investigated trimester‐specific alterations in plantar pressure, static and dynamic balance, and pain‐related functional interference in pregnant Taiwanese women.

**Methods:**

Eighty‐eight pregnant women were prospectively assessed across six gestational time points (8–36 weeks). Plantar pressure distribution and static balance were measured using a pressure plate system (FOOTPLATEC), and dynamic balance was evaluated using the timed up and go (TUG) test. Pain severity and its interference with daily activities were recorded using the Brief Pain Inventory. Foot oedema was assessed using a standardised grading system to evaluate trimester‐specific changes. Repeated‐measures ANOVA and Pearson correlation analyses were conducted.

**Results:**

Dynamic balance significantly declined across pregnancy, with TUG time increasing from 6.67 ± 1.11 s at 8–12 weeks to 7.24 ± 1.41 s at 33–36 weeks (*p* < 0.01, *η* ^2^ = 0.11). Medial midfoot pressure increased by 19% (from 102.56 ± 22.24 to 122.08 ± 29.93 kPa; *p* < 0.01). Mid‐pregnancy foot pain was inversely correlated with hallux (*r* = –0.17) and lesser toe pressures (*r* = –0.18; both *p* < 0.05), whereas heel pressure in late pregnancy was positively associated with low back pain (*r* = 0.23, *p* < 0.05). TUG performance was consistently correlated with interference in daily activities throughout gestation (*r* = 0.20–0.26, *p* < 0.05).

**Conclusion:**

Progressive redistribution of plantar loading and subtle balance changes were observed across gestation, which may contribute to discomfort and reduced mobility in some pregnant women. These findings support the importance of trimester‐specific biomechanical monitoring in prenatal care.

## 1 Introduction

The hormones relaxin and oestrogen, which increase markedly during pregnancy, contribute to ligamentous laxity throughout the pelvis and lower limbs, including the feet [1]. This hormonal effect, combined with gestational weight gain, significantly alters gait mechanics and the centre of gravity, resulting in postural instability and foot discomfort that elevate the risk of musculoskeletal injuries [2]. Hrvatin and Rugelj [3] reported that approximately 25% of pregnant women experience falls due to gait instability—a rate comparable to elderly populations [4]—underscoring the concomitant risks to maternal and foetal wellbeing.

In addition, fluid retention during pregnancy—particularly in the third trimester—often leads to lower limb oedema, which not only impairs mobility but also alters plantar pressure distribution and affects shoe fit [5, 6]. These physiological changes, alongside mechanical adaptations such as increased lumbar lordosis and thoracic kyphosis, reflect the body's compensation for the anterior weight shift during foetal growth [7, 8]. Empirical evidence further links gait instability to low back pain, with 60%–80% of women reporting foot or lumbar discomfort after 20 weeks' gestation [9, 10]. Such symptoms frequently impair mobility, sleep quality, and daily functioning, with 83.5% of women experiencing moderate‐to‐severe life interference in late pregnancy [11].

Concomitant with these systemic and mechanical changes are measurable alterations in foot morphology. Studies show that foot length and width progressively increase throughout the trimesters, although the total gain generally remains under 15 mm [12]. In contrast, ankle girth increases sharply only during the third trimester, primarily due to oedema. These changes—driven by the softening of connective tissue and increased weight—are associated with decreased arch height and expanded forefoot dimensions. Consequently, women often transition to larger footwear during mid to late pregnancy to accommodate swelling and alleviate pressure‐related foot pain [13, 14].

The foot is a tripartite structure comprising the forefoot (five metatarsals and phalanges), providing flexibility for propulsion and grip during gait [15, 16, 17]; the midfoot (cuboid, navicular and three cuneiform bones), which distributes load and attenuates pressure [18]; and the hindfoot (talus and calcaneus), governing stability in locomotion [16]. During pregnancy, physiological adaptations occur—including increased foot dimensions (length/width) and a larger plantar contact area [19]. These changes elevate peak pressures across the foot, modifying gait patterns and predisposing to pain or injury [20]. Concurrently, the anterior shift in the centre of gravity and added abdominal mass redistribute plantar loads: static measurements demonstrate reduced forefoot pressure but elevated hindfoot loading, whereas dynamic assessments reveal widespread pressure increases and diminished balance capacity [21, 22]. Such biomechanical shifts peak mid‐gestation, with excessive hindfoot pressure and pronation correlating strongly with lumbar pain [23]. Compounding this, ∼80% of women develop soft tissue oedema; when superimposed on weight gain, this exacerbates discomfort in pre‐existing joint pathologies and lowers the injury threshold for common foot conditions [24]. Moreover, pregnancy‐related oedema may alter plantar pressure distribution and compromise the accuracy of biomechanical assessments [25], highlighting the importance of its inclusion in related research.

In summary, the skeletal and muscular changes during pregnancy frequently result in low back pain and foot pain, which are common symptoms experienced by pregnant women. Although prior longitudinal studies have examined plantar contact area, plantar pressure, and balance adaptations throughout pregnancy, most have been conducted in Western populations. However, Asian women tend to exhibit distinct anthropometric characteristics, including shorter stature, narrower feet and lower longitudinal arches [26], which may influence plantar loading patterns and postural control responses. These morphological differences raise concerns about the applicability of existing findings to Asian populations and underscore the need for region‐specific evidence to inform clinical care. To address this gap, the present study adopts a longitudinal design to investigate the temporal changes in plantar contact area, plantar pressure and balance function from 8 to 36 weeks of gestation in Taiwanese women. The study also incorporates observations of pregnancy‐related oedema, foot pain and low back pain, as well as their impact on daily functioning. By combining standardised biomechanical assessments with symptom evaluations, this research study aims to provide an evidence‐base tailored to Asian pregnant women and to identify critical time windows for early intervention in foot health and postural support.

## 2 Method

### 2.1 Study Design and Ethical Considerations

This study adopted a longitudinal observational design and recruited a cohort of women who had recently confirmed their pregnancies at a research hospital in northern Taiwan. Ethical approval was obtained from Board of the Joint Institutional Review Board (JIRB) (Approval No. 16‐S‐023‐1). All participants received written information regarding the study and provided signed informed consent prior to enrolment.

### 2.2 Participants

The participants in this study were pregnant women. The inclusion criteria were as follows: women aged 18 years or older with a recently confirmed pregnancy; who were fully conscious; exhibited stable vital signs; had no history of smoking, alcohol, or drug addiction; were able to understand, speak, read, and write in Mandarin Chinese; were physically healthy with normal mobility and were willing to participate in all six treatments and complete the required questionnaires. Participants were excluded if they had any of the following: acute fractures, infections, inflammation, severe osteoporosis, or tumours; a history of lumbar spine or pelvic joint surgery or were currently receiving active conventional or traditional Chinese medical treatment or physiotherapy for foot‐related conditions.

### 2.3 Sample Size Calculation

The required sample size was estimated using G*Power 3.1.1 [27], based on a repeated measures ANOVA, following the methodology of Segal et al. [9]. The effect size was set at 0.15 (small to medium), with *α* = 0.05, power = 0.80, one group, and six repeated measures. The assumed correlation among repeated measures was 0.50, resulting in minimum required sample size of 72 participants. To account for potential attrition and enhance statistical precision, 88 participants were ultimately recruited, which exceeds the required number and ensures adequate power.

### 2.4 Procedure

This study was conducted in the outpatient clinics of a specialist obstetrics and gynaecology hospital, employing a consecutive sampling approach. Following eligibility screening, pregnant women between 8 and 12 weeks of gestation were recruited. The study's purpose, procedures and potential risks were explained in detail. Upon providing written informed consent, participants underwent data collection by trained research assistants. This included (1) demographic and obstetric history obtained via structured interviews; (2) completion of the Brief Pain Inventory through interviewer‐administered format, in which trained research assistants read each item aloud and clarified any questions as necessary and (3) anthropometric measurements carried out according to standardised protocols. Researchers demonstrated the procedures in advance for all biomechanical assessments and provided participants with practice trials. All measurements were performed by trained personnel between 08:00 and 11:00 a.m. to minimise the influence of diurnal variation in fluid distribution. Verbal instructions were standardised across all participants.

### 2.5 Measures

Demographic variables were recorded, including age, body weight, body mass index (BMI), number of foetuses, gestational weight gain, occupational type, parity (number of previous births ≥ 20 weeks' gestation), exercise habits and gestational age. A repeated‐measures design was employed, commencing at pregnancy confirmation (approximately 8–12 weeks' gestation) and continuing through to 36 weeks of gestation.

To facilitate comprehensive longitudinal evaluation, key outcome measures—including foot oedema grading, plantar pressure distribution, and pain assessment using the Brief Pain Inventory—were collected at six gestational time points: 8–12, 13–20, 21–24, 25–28, 29–32, and 33–36 weeks. This optimised assessment schedule allowed all data collection procedures to be completed within 20–30 min.

#### 2.5.1 Foot Oedema Grading

Foot oedema was assessed both visually and by palpation by trained researchers using a 0–3 grading scale (0 = no oedema, 1 = mild oedema, 2 = moderate oedema, 3 = severe oedema), adapted from widely used clinical classification methods [28].

#### 2.5.2 Foot Pressure and Static Balance Assessment

Plantar contact area, plantar pressure distribution and static balance were assessed using the FOOTPLATEC pressure plate (AMCube, UK) in conjunction with FootWork Pro for Windows software. Foot positioning on the platform was adjusted based on foot length to ensure consistent regional mapping across participants. The system is a capacitive‐sensor platform with a 5 mm‐thick plate comprising 2704 sensing elements arranged in a matrix layout, with a maximum acquisition frequency of 100 Hz. The system is pre‐calibrated by the manufacturer and does not require user calibration before data collection. Manual threshold adjustments are also unnecessary during routine use. Preventive maintenance, including calibration verification and sensor function checks, is performed every 3 months by an authorised engineer. Hardware inspections are conducted regularly to ensure signal integrity, and any anomalies are addressed in accordance with the service protocol. The FOOTPLATEC system have demonstrated high reliability, with intraclass correlation coefficients (ICCs) ranging from 0.72 to 0.99 under both static and dynamic conditions. These results support the use of the FOOTPLATEC system as a reliable tool for assessing plantar pressure and postural sway [29].

Participants stood with their feet on the FOOTPLATEC pressure plate in a relaxed bipedal stance (double‐limb support), with feet shoulder‐width apart, arms resting at their sides and gaze directed forward. All data were collected by trained research assistants who had received standardised training in foot placement and protocol execution. Two valid foot trials were conducted per session, and the mean regional pressures were recorded at the hallux, lesser toes, first metatarsal head, lesser metatarsals, medial and lateral midfoot and the heel. Additionally, the total plantar contact area (cm^2^) was automatically calculated by the system and used for analysis. This approach was based on pilot testing and is supported by study reporting acceptable reliability when using two trials with comparable pressure plate platforms [30]. The system recorded regional mean plantar pressure (kPa) rather than peak values across predefined foot zones.

For the static balance assessment, participants stood in a self‐selected stance to reflect their natural posture. One 20‐s trial was recorded for each condition (eyes open and eyes closed) while participants maintained a quiet stance. The system captured the centre of pressure (COP) trajectories, and sway amplitude was calculated in the anteroposterior (AP) and mediolateral (ML) directions. All data were processed using the manufacturer's software and exported for statistical analysis. This approach is commonly employed in postural sway assessments and was deemed appropriate based on our pilot testing and the low‐movement context of a static stance.

#### 2.5.3 Dynamic Balance Assessment

In addition, the timed up and go (TUG) test was employed to assess functional mobility. Participants were instructed to rise from a seated position on a standard‐height chair with armrests, walk 3 m, turn around, return and sit back down. For safety reasons, participants were permitted to use the armrests when standing. The time taken to complete this sequence was recorded using a stopwatch. The TUG test has demonstrated high reliability and validity in studies involving pregnant women [31].

#### 2.5.4 Brief Pain Inventory (BPI)

The Brief Pain Inventory (BPI), developed by Dr Charles S. Cleeland [32], was originally designed to assess cancer‐related pain. It has since been widely adopted for evaluating both the severity of pain and its impact on daily functioning. In this study, the BPI was used to assess the severity and interference of two common pregnancy‐related symptoms: foot pain and low back pain. Participants were asked to report the intensity of pain specifically in the feet and lower back, as well as the extent to which the pain interfered with daily life activities, such as walking, sleeping, mood, and general activity. The BPI comprises 17 items and assesses two core dimensions: pain severity and pain‐related interference. Each item is ranging from 0 (no pain/no interference) to 10 (worst imaginable pain/complete interference). Scores for pain severity (range: 0–40) and interference (range: 0–70) were calculated as the sum of the relevant items. Previous studies have demonstrated strong internal consistency of the BPI in populations with chronic noncancer pain, with Cronbach's alpha values reported as 0.85 for severity and 0.88 for interference domains [33].

### 2.6 Statistical Analysis

Data were analysed using SPSS version 26.0 for Windows. Descriptive statistics were first generated to summarise participant characteristics. Pearson's correlation analyses were used to examine trimester‐specific associations between foot pain, low back pain, interference with daily activities, plantar pressure distribution, and balance metrics across the six gestational time points. Longitudinal changes were analysed using repeated‐measures analysis of variance (ANOVA), with Bonferroni post hoc tests applied to identify significant differences between trimesters in plantar contact area, foot oedema grading, plantar pressure patterns and balance performance.

Attrition occurred at several interim stages, resulting in missing data at some gestational time points. Missing values were addressed using linear interpolation, which estimated intermediate values based on adjacent available data [34]. All statistical tests were two‐tailed, with significance set at *p* < 0.05.

## 3 Results

A total of 326 pregnant women were screened for eligibility at their initial prenatal visit. Of these, 88 women met all inclusion criteria and provided written informed consent to participate in this longitudinal study. Longitudinal data were collected at six gestational time points, with attrition occurring at several interim stages (*n* = 2 at 13–20 weeks, *n* = 8 at 21–24 weeks, *n* = 15 at 25–28 weeks, and *n* = 8 at 29–32 weeks). Complete data sets were available for all participants at the baseline (8–12 weeks) and final (33–36 weeks) assessments. Although no participants formally withdrew from the study, intermittent nonresponse occurred due to missed follow‐up appointments or scheduling difficulties. The number of valid responses per time point ranged from 73 to 88, representing an attrition rate of 2.27%–17.05% across gestational time points.

The ages of the pregnant women ranged from 19 to 44 years, with a mean age of 32.26 years (SD = 5.30). The mean gestational age at recruitment was 10.39 weeks. Participants had an average height of 160.44 cm (SD = 4.67). The majority were employed (75%), reported no history of medical conditions (96.6%) and most had not participated in regular physical activity (80.7%). In terms of obstetric history, 54.5% were primiparous. The average pre‐pregnancy weight was 54.13 kg (SD = 8.34). By 36 weeks of gestation, the mean weight had increased to 65.54 kg (SD = 9.09), corresponding to an average gestational weight gain of 11.41 kg (SD = 4.24).

The severity of foot oedema showed a notable progression throughout the course of pregnancy. In early pregnancy (8–12 weeks), 12.59% of participants presented with mild oedema (grade 1+), whereas no cases of moderate oedema (grade 2+) were recorded. By mid‐pregnancy, the prevalence of 1+ oedema rose to 28.47%, and 2.08% of women exhibited 2+ oedema. In late pregnancy, oedema rates peaked, with 30.00% of participants presenting with 1+ oedema and 10.91% with 2+ oedema. Collectively, these findings indicate that 40.91% of pregnant women experienced clinically significant oedema during the third trimester. A chi‐square test revealed a statistically significant increase in oedema severity across gestational stages (*χ* ^2^ = 24.03, *p* < 0.05). The plantar contact area also increased over the course of pregnancy. Compared to baseline values recorded at 8–12 weeks' gestation, the plantar contact area of the left foot increased by 0.51% ± 0.70 and further to 0.55% ± 0.68 by 36 weeks. The right foot demonstrated a similar pattern, with an increase of 0.51% ± 0.72 and 0.56% ± 0.63 by late pregnancy.

Longitudinal analysis revealed significant progression of pregnancy‐related symptoms across all measured parameters (Table 1). Body weight and BMI demonstrated continuous increases between all six gestational time points (*p* < 0.05), whereas plantar contact area showed significant differences between early (Times points 1–3) and late gestation (Times points 4–6). Pain symptoms exhibited particularly marked progression: foot pain severity increased nearly 12‐fold from early to late pregnancy (0.27 ± 1.05 at 8–12 weeks vs. 3.21 ± 6.77 at 33–36 weeks; *p* < 0.01), low back pain scores nearly doubled (3.55 ± 6.07 to 6.32 ± 8.74; *p* < 0.05) and pain‐related life interference scores almost tripled (3.95 ± 7.78 to 11.01 ± 17.27; *p* < 0.01). Post‐hoc analyses identified distinct progression patterns, with foot pain showing a delayed onset, and significant differences observed only between gestational time points 1 and 3 compared to time point 6. Life interference increased progressively, with gestational time points 1 and 2 differing from points 4 to 6, and time point 2 specifically differing from point 5. These findings highlight three key progression trajectories: continuous (weight/BMI), delayed‐onset (foot pain) and mid‐to‐late gestation escalation (plantar contact area and life interference). Comprehensive data are presented in Table 1.

**TABLE 1:** Changes in body weight, BMI, plantar contact area, and BPI.

Characteristics	Prior to conception	8–12 weeks (*n* = 88)	13–20 weeks (*n* = 86)	21–24 weeks (*n* = 80)	25–28 weeks (*n* = 73)	29–32 weeks (*n* = 80)	33–36 weeks (*n* = 88)	*F/χ* ^2^	*η* ^2^
	M ± SD	M ± SD	M ± SD	M ± SD	M ± SD	M ± SD	M ± SD		
Body weight	54.13 ± 8.34	55.32 ± 8.24	58.80 ± 8.90	60.72 ± 9.00	62.29 ± 8.56	63.71 ± 9.22	65.54 ± 9.09	273.35**	0.82
BMI	20.99 ± 2.88	21.31 ± 2.54	22.63 ± 2.72	23.38 ± 2.74	24.00 ± 2.74	24.55 ± 2.86	25.26 ± 2.85	260.98**	0.82
Increased plantar contact area									
Left	—	0.51 ± 0.70	0.52 ± 0.72	0.52 ± 0.70	0.53 ± 0.71	0.54 ± 0.67	0.55 ± 0.68	38.28**	0.39
Right	—	0.51 ± 0.72	0.52 ± 0.72	0.53 ± 0.65	0.54 ± 0.63	0.55 ± 0.59	0.56 ± 0.63	40.27**	0.40
Foot oedema grading								24.03**	
0		78	77	65	55	57	55		
1		10	9	13	17	22	24		
2		0	0	2	1	1	9		
3		0	0	0	0	0	0		
BPI									
Foot pain	—	0.27 ± 1.05	0.73 ± 2.73	0.97 ± 3.71	1.50 ± 4.29	2.17 ± 5.41	3.21 ± 6.77	5.74**	0.09
Low back pain	—	3.55 ± 6.07	4.08 ± 6.53	5.28 ± 7.67	5.47 ± 7.58	6.43 ± 8.27	6.32 ± 8.74	2.90*	0.05
Life interference	—	3.95 ± 7.78	4.78 ± 8.90	7.42 ± 13.32	8.56 ± 12.07	10.20 ± 13.53	11.01 ± 17.27	6.90**	0.11

Abbreviations: BM, body mass index; BPI, brief pain inventory (foot pain, low back pain, life interference).

^*^ *p* < 0.05.

^**^ *p* < 0.01.

The plantar pressure distribution demonstrated notable temporal variations throughout gestation. Post hoc analyses revealed that pressure beneath the medial midfoot exhibited an early rise, with significant increases observed in weeks 13–20 (110.81 ± 22.24 kPa), 21–24 (117.98 ± 26.84 kPa), 25–28 (119.82 ± 27.45 kPa) and 29–32 (114.94 ± 29.78 kPa) compared to weeks 8–12 (102.56 ± 22.24 kPa), peaking at 33–36 weeks (122.08 ± 29.95 kPa, *F* = 9.23, *p* < 0.01, *η* ^2^ = 0.10). Forefoot pressures indicated that hallux pressure at 25–28 weeks (222.94 ± 62.65 kPa) was significantly higher than at 8–12 weeks (198.47 ± 64.34 kPa, *p* < 0.05), whereas lesser toes pressure peaked at 21–24 weeks (170.79 ± 36.12 kPa), significantly exceeding both baseline (8–12 weeks: 157.89 ± 40.34 kPa) and late gestation (29–32 weeks: 164.25 ± 32.95 kPa, *F* = 3.66, *p* < 0.05, *η* ^2^ = 0.04). Heel pressure reached its maximum at 21–24 weeks (222.45 ± 32.48 kPa, *p* < 0.05, *η* ^2^ = 0.03), but post hoc tests showed no significant pairwise differences in other periods.

For balance assessments, post hoc analyses of the TUG test revealed significant deterioration in dynamic balance during late pregnancy. Specifically, performance at weeks 29–32 (7.08 ± 1.20 s) was significantly slower compared to weeks 8–12 (6.67 ± 1.11 s), whereas weeks 33–36 (7.24 ± 1.41 s) showed further slowing relative to both weeks 13–20 (6.84 ± 1.23 s) and 21–24 (6.73 ± 0.99 s) (*F* = 7.15, *p* < 0.01, *η* ^2^ = 0.11). In contrast, COP measures, including both eyes‐open and eyes‐closed conditions, showed no statistically significant changes across all gestational periods (Table 2).

**TABLE 2:** Changes in plantar pressure, static and dynamic balance assessment.

Characteristics	8–12 weeks	13–20 weeks	21–24 weeks	25–28 weeks	29–32 weeks	33–36 weeks	*F*	*η* ^2^
	M ± SD	M ± SD	M ± SD	M ± SD	M ± SD	M ± SD		
Plantar pressure								
Hallux pressure	198.47 ± 64.34	206.18 ± 71.98	218.70 ± 67.10	222.94 ± 62.65	202.53 ± 56.75	214.61 ± 71.99	3.35*	0.04
Lesser toes pressure	157.89 ± 40.34	162.92 ± 40.08	170.79 ± 36.12	169.61 ± 36.73	164.25 ± 32.95	158.35 ± 35.59	3.66*	0.04
First metatarsal pressure	211.07 ± 42.85	220.59 ± 52.77	213.14 ± 35.19	217.01 ± 36.60	222.55 ± 40.69	225.76 ± 57.95	1.96	0.02
Lateral metatarsal pressure	202.39 ± 52.80	202.55 ± 39.20	206.90 ± 42.29	201.05 ± 35.87	202.66 ± 39.43	198.33 ± 39.87	2.37	0.02
Medial midfoot pressure	102.56 ± 22.24	110.81 ± 28.72	117.98 ± 26.84	119.82 ± 27.45	114.94 ± 29.78	122.08 ± 29.95	9.23**	0.10
Lateral midfoot pressure	130.69 ± 34.72	128.59 ± 30.64	138.58 ± 33.51	140.14 ± 35.79	135.94 ± 33.27	135.94 ± 33.27	0.30*	0.03
Heel pressure	210.07 ± 49.46	214.21 ± 36.14	222.45 ± 32.48	215.74 ± 29.85	212.96 ± 29.60	218.14 ± 24.65	2.79*	0.03
Static balance assessment (COP)								
Eyes open, AP	21.07 ± 8.79	22.22 ± 8.79	23.16 ± 9.94	24.64 ± 14.42	26.04 ± 16.98	27.90 ± 13.41	2.15	0.07
Eyes open, ML	14.16 ± 4.13	15.62 ± 5.85	15.65 ± 6.63	15.52 ± 6.27	15.56 ± 6.01	17.57 ± 5.79	1.34	0.04
Eyes closed, AP	24.04 ± 8.99	27.23 ± 13.56	25.99 ± 8.36	27.30 ± 9.75	28.18 ± 9.514	27.62 ± 13.23	26.00	0.14
Eyes closed, ML	13.51 ± 4.49	15.40 ± 5.88	14.88 ± 5.80	15.77 ± 6.32	16.05 ± 6.07	16.08 ± 5.26	1.33	0.04
TUG	6.67 ± 1.11	6.84 ± 1.23	6.73 ± 0.99	6.81 ± 1.15	7.08 ± 1.20	7.24 ± 1.41	7.15**	0.11

*Note:* Plantar pressure values are reported in kilopascals (kPa).

Abbreviations: AP, anteroposterior displacements; COP, centre of pressure: sway amplitude in millimetres (mm); ML, mediolateral displacements; TUG, timed up and go test: time in seconds (s).

^*^ *p* < 0.05.

^**^ *p* < 0.01.

Longitudinal analysis revealed trimester‐specific biomechanical relationships: During the first trimester, plantar pressures exhibited no significant correlations with foot pain or life interference. However, dynamic balance already demonstrated weak associations with foot pain (*r* = 0.26, *p* < 0.01), low back pain (*r* = 0.20, *p* < 0.05) and life interference (*r* = 0.23, *p* < 0.05). By the second trimester, foot pain developed modest negative correlations with hallux pressure (*r* = −0.17, *p* < 0.05) and lesser toes pressure (*r* = −0.18, *p* < 0.05), whereas life interference began linking to dynamic balance (*r* = 0.20, *p* < 0.05). These associations strengthened in the third trimester, with heel pressure showing a positive correlation with low back pain (*r* = 0.23, *p* < 0.05). Additionally, dynamic balance exhibited stronger links with foot pain (*r* = 0.23, *p* < 0.05) and life interference (*r* = 0.26, *p* < 0.001). Notably, static balance measures (AP/ML sway) demonstrated high internal consistency across trimesters (eyes‐open: *r* = 0.54–0.56, *p* < 0.01; eyes‐closed: *r* = 0.54–0.58, *p* < 0.01) but remained unrelated to pain symptoms (Tables 3, 4, 5).

**TABLE 3:** Correlation between foot pain, lower back pain, life interference, plantar pressure, static and dynamic balance during the first trimester.

Variables	1	2	3	4	5	6	7	8	9	10	11	12	13	14
1. Foot pain														
2. Low back pain	0.45^**^													
3. Life interference	0.28^**^	0.59^**^												
4. Hallux pressure	0.07	−0.01	0.35											
5. Lesser toes pressure	0.57	−0.51	0.62	0.65^**^										
6. First metatarsal pressure	−0.01	0.08	0.07	0.51^**^	0.42^**^									
7. Lateral metatarsal pressure	−0.06	0.06	0.11	0.47^**^	0.71^**^	0.52^**^								
8. Medial midfoot pressure	−0.05	−0.02	0.02	0.36^**^	0.75^**^	0.54^**^	0.72^**^							
9. Lateral midfoot pressure	0.12	0.08	0.14	0.40^**^	0.70^**^	0.45^**^	0.78^**^	0.76^**^						
10. Heel pressure	−0.04	0.10	0.07	0.42^**^	0.57^**^	0.45^**^	0.57^**^	0.50^**^	0.50^*^					
11. Eyes open AP	−0.04	−0.04	−0.06	−0.05	−0.04	−0.18	−0.01	−0.06	−0.03	−0.15				
12. Eyes open, ML	−0.03	−0.02	−0.09	0.09	−0.08	0.08	−0.02	−0.07	0.00	−0.13	0.54^**^			
13. Eyes closed, AP	−0.07	−0.05	0.05	−0.03	0.05	−0.20	0.08	−0.11	0.07	−0.05	0.50^**^	0.32^**^		
14. Eyes closed, mediolateral ML	−0.12	−0.01	−0.07	0.12	0.10	−0.02	−0.01	−0.06	0.05	0.09	0.20	0.28^**^	0.55^**^	
15. TUG	0.26^**^	0.20^*^	0.23^*^	−0.05	−0.06	−0.11	−0.03	0.05	0.10	−0.10	0.07	0.05	0.20	−0.08

Abbreviations: AP, anteroposterior displacements; ML, mediolateral displacements; TUG, timed up and go test: time in seconds (s).

^*^ *p* < 0.05.

^**^ *p* < 0.01.

**TABLE 4:** Correlation between foot pain, low back pain, life interference, plantar pressure, and gait balance in the second trimester.

Variables	1	2	3	4	5	6	7	8	9	10	11	12	13	14
1. Foot pain														
2. Low back pain	0.32^**^													
3. Life interference	0.54^**^	0.24^**^												
4. Hallux pressure	−0.17^*^	−0.14	−0.18											
5. Lesser toes pressure	−0.18^*^	−0.03	−0.17	0.42^**^										
6. First metatarsal pressure	−0.15	−0.05	0.14	0.19^*^	0.12									
7. Lateral midfoot pressure	−0.09	0.01	−0.11	0.16	0.17^*^	0.19^*^								
8. Medial midfoot pressure	−0.09	−0.03	−0.11	−0.08	0.10	0.38^**^	0.11							
9. Lateral foot pressure	−0.03	0.06	−0.05	0.15	0.09	0.18^*^	0.45^**^	0.27^**^						
10. Heel pressure	−0.13	.‐02	0.10	0.12	0.07	−0.01	0.23^**^	0.09	0.24^**^					
11. Eyes open AP	0.03	0.00	0.17	−0.12	−0.04	−0.16	−0.03	0.02	−0.16	0.05				
12. Eyes open, ML	−0.00	−0.11	0.05	−0.17	−0.09	−0.05	0.04	−0.01	−0.03	0.04	0.56^**^			
13. Eyes closed, AP	−0.09	−0.04	0.05	0.07	−0.01	−0.03	−0.01	−0.13	−0.02	−0.04	0.25^**^	0.25^**^		
14. Eyes closed, mediolateral ML	0.04	0.02	−0.04	−0.01	−0.05	−0.04	−0.04	−0.14	−0.03	−0.05	0.35^**^	0.45^**^	0.58^**^	
15. TUG	0.08	0.07	0.20^*^	−0.18^*^	−0.17^*^	−0.13	−0.11	0.09	0.07	−0.12	−0.02	0.04	0.01	0.01

Abbreviations: AP, anteroposterior displacements; ML, mediolateral displacements; TUG, timed up and go test: time in seconds (s).

^*^ *p* < 0.05.

^**^ *p* < 0.01.

**TABLE 5:** Correlation between foot pain, lower back pain, life interference, plantar pressure, and gait balance in the third trimester.

Variables	1	2	3	4	5	6	7	8	9	10	11	12	13	14
1. Foot pain														
2. Low back pain	0.47^**^													
3. Life interference	0.61^**^	0.88^**^												
4. Hallux pressure	−0.00	0.08	0.05											
5. Lesser toes pressure	−0.02	0.04	−0.04	0.65^**^										
6. First metatarsal pressure	−0.01	0.08	0.06	0.51^**^	0.42^**^									
7. Lateral metatarsal pressure	−0.01	0.12	0.09	0.47^**^	0.71^**^	0.52^**^								
8. Medial midfoot pressure	−0.01	0.03	0.01	0.36^**^	0.75^**^	0.54^**^	0.72^**^							
9. Lateral midfoot pressure	0.05	0.05	0.04	0.40^**^	0.70^**^	0.45^*^	0.77^**^	0.76^**^						
10. Heel pressure	0.08	0.23^*^	0.22	0.42^**^	0.57^**^	0.45^**^	0.57^**^	0.50^**^	0.50^**^					
11. Eyes open AP	−0.12	−0.11	−0.11	−0.05	−0.05	−0.18	−0.01	−0.06	−0.03	−0.15				
12. Eyes open, ML	−0.04	−0.10	−0.10	0.09	−0.08	0.07	−0.02	−0.06	0.00	−0.12	0.54^**^			
13. Eyes closed, AP	−0.04	−0.04	−0.02	−0.03	0.05	−0.02	0.09	−0.11	0.07	−0.05	0.50^**^	0.32^**^		
14. Eyes closed, mediolateral ML	0.10	0.07	0.07	0.12	0.10	−0.02	−0.07	−0.06	0.05	0.09	0.20	0.28^**^	0.54^**^	
15. TUG	0.23^*^	0.19	0.26^**^	−0.05	−0.06	−0.11	−0.03	0.05	0.10	−0.10	0.07	0.05	0.11	−0.08

Abbreviations: AP, anteroposterior displacements; ML, mediolateral displacements; TUG, timed up and go test: time in seconds (s).

^*^ *p* < 0.05.

^**^ *p* < 0.01.

## 4 Discussion

### 4.1 General Changes in Plantar Contact Area

This study identified significant longitudinal changes in plantar contact characteristics, plantar pressure distribution and balance performance during pregnancy. Our cohort exhibited a modest mean increase in plantar contact area (0.04%) based on pressure plate analysis. Several studies have reported significant increases in plantar contact area during pregnancy—particularly in the third trimester—although most do not provide specific percentage values [35]. Importantly, an increase in contact area does not directly indicate an anatomical increase in foot length or width, rather it reflects functional adaptations such as arch flattening and altered plantar loading. These changes are consistent with gestational adaptations, including ligamentous laxity, weight gain, and postural adjustments [36]. Supporting this interpretation, Tsung et al. [37] reported that contact area increased under progressive loading conditions without proportional changes in foot dimensions, emphasising that plantar contact area is a measure of weight‐bearing morphology rather than static anatomical size.

### 4.2 Patterns of Oedema and Ethnic Variation

Interestingly, the prevalence of pregnancy‐related oedema followed an atypical pattern: mid‐gestation prevalence (28.47%) exceeded prior reports in other Asian populations, such as Pakistani women (10.3%) [38], whereas late‐pregnancy rates (40.91%) were lower than those observed in Pakistan (65.9%) [39] In late pregnancy, our prevalence was close to that reported in Western populations, such as Bulgarian women (∼40%) during the second and third trimesters [40]. However, although oedema prevalence among Bulgarian women showed little difference between mid‐ and late pregnancy, our cohort demonstrated a marked increase across gestation, suggesting population‐specific temporal patterns. These discrepancies may reflect ethnic variations in physiological adaptation, differences in oedema assessment methods (clinical evaluation vs. self‐report), or the relatively low mean BMI (23.4 kg/m^2^) in population sample.

### 4.3 Temporal Redistribution of Plantar Pressure

Our findings revealed temporally distinct pressure peaks across different plantar regions, indicating region‐specific adaptations rather than a uniform increase in plantar pressure. Heel pressure peaked earlier at between 21 and 24 weeks of gestation, whereas hallux pressure continued to rise, reaching its maximum between 25 and 28 weeks. Notably, medial midfoot pressure increased progressively from the second trimester and showed significant differences across nearly all time points compared to the baseline, which may indicate progressive arch flattening and increased midfoot loading.

These staggered peaks likely represent a sequential adaptation strategy: rearfoot adjustments may occur earlier to manage emerging postural instability, followed by compensatory shifts in forefoot and midfoot loading as gestational weight gain progresses. This interpretation aligns with previous evidence suggesting that plantar pressure redistribution is influenced by gestational weight, ligamentous laxity, and changes in foot posture [41, 42].

Unlike studies reporting generalised increases in plantar pressure during the third trimester [43, 44], our results emphasise the value of segmental analysis in revealing the temporal specificity of biomechanical adaptations. For instance, Western studies have largely described a more linear progression of plantar pressure shifts. Masłoń et al. [45] observed that increases in maternal weight and abdominal girth in Polish women were accompanied by progressive rises in midfoot and forefoot loading with corresponding reductions at the rearfoot, whereas Martínez‐Martí et al. (2019) reported that Spanish women showed a forward shift of plantar loading across gestation, with forefoot pressure significantly associated with low back pain [23]. In contrast, our Taiwanese cohort demonstrated temporally staggered pressure peaks across regions, suggesting population‐specific adaptation strategies.

### 4.4 Associations Between Plantar Pressure, Balance, and Related Pain

In our correlational analysis, foot pain showed distinct associations with segmental plantar pressure changes during pregnancy. Specifically, it was significantly correlated with reduced plantar pressure at the hallux and lesser toes, suggesting that pregnant women may subconsciously modify their gait mechanics to reduce forefoot loading. This may reflect a shift away from normal toe‐off propulsion, potentially adopting a ‘lower gear’ walking strategy characterised by slower movement and reduced forefoot push‐off. Such adaptations could serve to alleviate discomfort and enhance postural stability during mid‐gestation [46].

In the third trimester, the associations between plantar pressure and symptom burden became more pronounced. Specifically, heel pressure was positively correlated with low back pain (*r* = 0.23, *p* < 0.05), suggesting that increased rearfoot loading may reflect compensatory strategies in response to anterior postural shifts. This shift in pressure distribution could contribute to increased lumbar strain as maternal weight and abdominal protrusion intensify near term [47].

Additionally, dynamic balance performance showed significant correlations with foot pain (*r* = 0.23, *p* < 0.05) and life interference (*r* = 0.26, *p* < 0.001). Although the mean increase in TUG completion time between mid‐ and late pregnancy in our cohort was only 0.51 s, this change did not exceed the minimal detectable change of 1.16 s [30], indicating that the observed difference is unlikely to reflect clinically meaningful deterioration. Rather than pathological impairment, this modest increase in TUG time likely reflects normal gestational adaptations, such as increased maternal weight [48], reduced postural control, and altered gait mechanics.

### 4.5 Study Limitations

Several limitations must be acknowledged. Although baseline and final data were complete, intermediate timepoints experienced 2.27%–17.05% attrition, which may affect the reliability of trajectory modelling. In addition, the use of a convenience sample from a single teaching hospital limits the generalisability of the findings. Stratification by parity, assessment of occupational physical demands, and postpartum follow‐up were not conducted. Furthermore, foot length and width were not directly measured at each gestational time point; therefore, interpretations of changes in foot size are based solely on indirect indicators, such as plantar contact area and pressure distribution, which reflect functional rather than anatomical adaptations. Additionally, foot posture was not assessed using a standardised clinical tool, such as the foot posture index. Although plantar pressure mapping provides insights into foot function, the absence of direct foot posture classification limits the ability to associate pressure changes with specific morphological alterations, such as arch profile or pronation status.

### 4.6 Clinical Implications

Pregnancy‐induced changes in the foot can significantly alter plantar pressure distribution and postural control, resulting in foot and lower back discomfort that may impair functional ability and quality of life. The observed correlations between plantar pressure redistribution, foot pain and mobility performance highlight the importance of early identification of musculoskeletal issues during gestation. Healthcare practitioners should incorporate systematic podiatric assessments into routine prenatal care and provide evidence‐based interventions to support maternal walking function and alleviate pregnancy‐related complaints throughout gestation. Findings from this study also reinforce clinical caution regarding the prescription of customised orthotics during pregnancy, as foot structure and loading patterns remain dynamic. Segal et al. [49] demonstrated that arch collapse can progress throughout pregnancy despite the use of customised insoles. Therefore, delaying orthotic prescription until foot morphology stabilises postnatally may be more appropriate.

### 4.7 Directions for Future Research

This study offers novel temporal insights into foot biomechanics in Asian pregnant women through standardised longitudinal assessments. It highlights distinct patterns of oedema prevalence and unique plantar pressure trajectories compared with Western populations, emphasising the importance of ethnicity‐specific research in maternal biomechanics. Future studies should incorporate more diverse populations, standardised assessments of foot posture, extended observation periods and postpartum follow‐up to more comprehensively capture the full trajectory of biomechanical changes during and after pregnancy.

## 5 Conclusion

Pregnancy‐induced changes in the foot lead to temporal and region‐specific redistributions of plantar pressure, which are associated with balance performance, foot pain and low back pain. These findings provide novel longitudinal insights into maternal biomechanics in an Asian population, highlighting functional adaptations rather than static anatomical changes.

## Author Contributions

**Meei‐Ling Gau:** conceptualization, methodology, formal analysis, funding acquisition, writing – original draft, writing – review and editing. **Hui‐Mei Hsu:** data curation, investigation, writing – original draft. **Li‐Li Chen:** investigation, writing – review and editing. **Tzu‐Ling Chen:** investigation, writing – review and editing. **Wan‐Lin Pan:** supervision, formal analysis, writing – original draft, writing – review and editing.

## Funding

This work was supported by the National Science and Technology Council, Taiwan (NSTC 114‐2314‐B‐227‐009), and previously by the Ministry of Science and Technology, Taiwan (MOST 106‐2314‐B‐227‐005, MOST 107‐2314‐B‐227‐003, and MOST 108‐2314‐B‐227‐006).

## Ethics Statement

This study was approved by the Joint Institutional Review Board (JIRB) (Approval No. 16‐S‐023‐1). Written informed consent was obtained from all participants prior to enrolment.

## Consent

Written informed consent was obtained from all participants prior to enrolment.

## Conflicts of Interest

The authors declare no conflicts of interest.

## Data Availability

The datasets used and analysed during the current study are available from the corresponding author on reasonable request.
